# Priorities for Bolstering Public Health Resilience in the Context of Climate Change in Dominica and Puerto Rico

**DOI:** 10.5334/aogh.3876

**Published:** 2022-07-27

**Authors:** Hannah H. Covert, Lissa Fortes Soares, Firoz Abdoel Wahid, Teddy Allen, Zack Guido, David Johnson, Roché Mahon, Pablo Méndez-Lázaro, Mya Sherman, Sylvester St. Ville, Adrian Trotman, Maureen Y. Lichtveld

**Affiliations:** 1 Department of Environmental Health Sciences, School of Public Health and Tropical Medicine, Tulane University, New Orleans, Louisiana, US; 2 Department of Environmental and Occupational Health, School of Public Health, University of Pittsburgh, Pittsburgh, Pennsylvania, US; 3 Caribbean Institute for Meteorology and Hydrology, St. James, Barbados; 4 Arizona Institute for Resilience Environments and Societies and the School of Natural Resources and Environment, The University of Arizona, Tucson, Arizona, US; 5 Ministry of Health, Wellness and New Health Investment, Roseau, Dominica; 6 Envronmental Health Department, Graduate School of Public Health, University of Puerto Rico-Medical Campus, San Juan, Puerto Rico

**Keywords:** environmental scan, climate and health, Caribbean, Dominica, Puerto Rico, small island developing states (SIDS)

## Abstract

Caribbean small island developing states are highly exposed to climate change impacts. Incorporating weather and climate information into public health decisions can promote resilience to climate change’s adverse health effects, but regionally it is not common practice. We implemented a project to enhance dialogue between climate and public health specialists in Puerto Rico and Dominica. First, we conducted environmental scans of public health vulnerability in the context of weather and climate for both islands. Then, we convened stakeholders to discuss the scan results and identify priorities for climate and health. A shared priority was increasing climate and health knowledge; thus, we developed several educational initiatives. In this viewpoint, we discuss our process for conducting environmental scans, building capacity and partnerships, and translating knowledge-to-action around climate and health.

## Introduction

The Caribbean region experiences an average of 14 annual tropical storms and hurricanes, some of which harm human and environmental systems [1]. Much less attention is paid to other weather and climate events in the region, such as floods, drought, extreme heat, and African dust storms [2,3,4]. With climate change, expected and familiar climate patterns have been altered, including the timing, intensity and frequency of climate events [5,6] Increased health risks from climate change include vector- and water-borne diseases, heat-related conditions, respiratory disorders, food and water insecurity, mental health disorders, and injury and death from extreme events [7,8]. Critical health care infrastructure and systems are threatened by climate hazards and there is a push to develop climate resilient health systems [9,10].

The 28 small island developing states (SIDS) in the Caribbean are among the most globally vulnerable to climate change and its adverse health consequences [6,8,11]. Many Caribbean SIDS rely economically on environment-dependent sectors such as tourism, agriculture, and fisheries [6]. Health systems in the region are underfunded, face a shortage of health professionals, and have limited infrastructure [12]. The region has a historic burden of health and social disparities [13].

Across Caribbean countries, a lack of resources and specialized knowledge as well as siloed agencies and work processes inhibit public health practitioners from making full use of available sector-specific climate products, such as the quarterly Caribbean Health Climatic Bulletin and other generic weather and climate products, to manage health risks and promote climate resiliency [2,4,14,15]. These products are supported by national and regional organizations such as the National Meteorological and Hydrological Services, the Caribbean Institute for Meteorology and Hydrology (CIMH), the Caribbean Meteorological Organization (CMO), the Caribbean Public Health Agency (CARPHA), the Pan American Health Organization (PAHO), the Climate Studies Group at the University of the West Indies-Mona, and the US National Oceanic and Atmospheric Administration. Despite the high capacity for climate and weather services, public health decision-making in most Caribbean nations does not incorporate climate information. Improving dialogue and connections between climate and public health specialists through knowledge networks, forums, and communities of practice can enhance connecting climate information to decision-making for public health [2,4,14,16,17].

**Figure 1:**
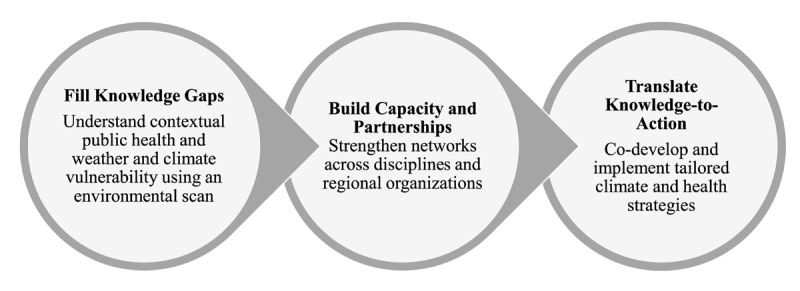
Project roadmap.

We implemented a project to foster dialogue and connections between climate and public health specialists in Puerto Rico and Dominica. First, we conducted environmental scans of public health vulnerability in the context of weather and climate for both sites. We subsequently convened in-country stakeholder workshops where climate and public health specialists discussed the scan results, and co-produced priority actions to address climate and health. One action identified at both workshops was the need to increase knowledge and awareness about climate and health. Consequently, we convened educational activities to address this gap. In this viewpoint, we describe each country’s priorities and discuss our process for conducting environmental scans to fill knowledge gaps, building capacity and partnerships, and translating knowledge-to-action around climate and health (Figure 1).

## Environmental scan

**Figure 2:**
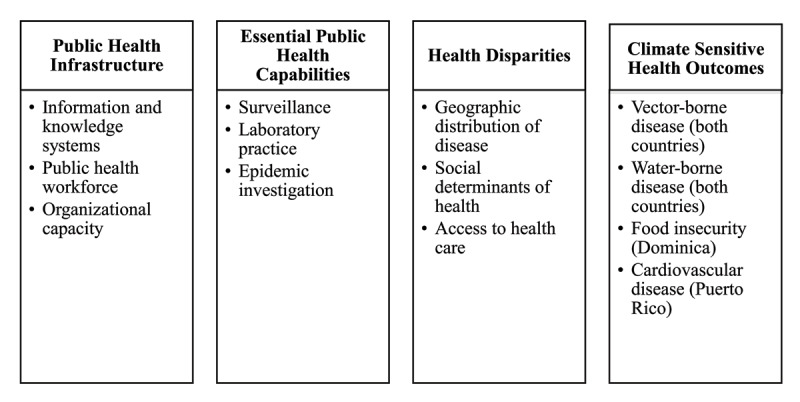
Environmental scan public health domains.

The environmental scan is a tool to collect data and information for public health planning and decision-making. The purpose is “to understand context; collect information; and identify resources, links, and gaps” (p. 1) [18]. Scans are useful in complex situations, such as climate change [19]. Information is collected from internal and external sources (e.g. peer-reviewed literature, reports, policy documents) through literature reviews, surveys, interviews, and observations, among others [19]. We conducted environmental scans for Puerto Rico and Dominica to assess four public health domains (Figure 2). The defining characteristics are fixed for public health infrastructure, essential capabilities, and health disparities, but climate-sensitive health concerns vary by context. Climate-sensitive health concerns are those conditions that are exacerbated by changes in climate, such as higher temperatures, poorer air quality, or the increasing frequency of extreme weather.

## Stakeholder workshops

We presented the environmental scan results at 1.5-day workshops in San Juan, Puerto Rico (March 2019) and Roseau, Dominica (May 2019) to stakeholders in the public health and meteorological sectors from non-profit organizations, academia, and government agencies. At each workshop, 19 participants discussed the scan results and identified island-specific priority actions for addressing climate and health. The workshops created space for collaborative learning and networking between siloed professionals. At the time of the workshops in 2019, Dominica and Puerto Rico were recovering from the devastating impacts of Hurricane Maria, which occurred in September 2017. This disaster was a common point of discussion, emphasizing the interconnectedness and urgency of addressing climate and public health.

### Puerto Rico

Puerto Rico, a U.S. territory of 3.2 million people, comprises 8.9 thousand square kilometers [20]. Puerto Ricans are US citizens, almost all are Hispanic or Latino (98%) and speak Spanish (95%) [21]. The environmental scan highlighted challenges in each of the four domains. From an infrastructure perspective, Puerto Rico’s mixed public-private health care services are constrained by a lack of critical health facilities, a shortage of physicians, and an aging population [22]. Essential capabilities addressing vector control and health surveillance are supported by the US Centers for Disease Control and Prevention. Although Puerto Rico is designated as a high-income economy, the poverty rate is 44% (compared to the US average of 11%) [20,21,23]. Health disparities include high rates of chronic disease (e.g., cardiovascular disease, asthma, and hypertension), impacting disaster preparedness and response [22]. The key weather factors are heat, hurricanes, drought, precipitation, and African dust events. Climate-sensitive health concerns include respiratory illnesses, cardiovascular disease, and vector-borne diseases (e.g., leptospirosis dengue, chikungunya, and Zika) [24].

### Dominica

Dominica is 800 square kilometers and has a population of approximately 72 000 people, of which 85% are African descent, 9% are mixed race, and 4% are Indigenous. Dominicans speak English and French patois [25,26]. The country’s infrastructure challenges include brain drain among its health professionals and a primarily paper-based health information system [27]. In terms of essential capabilities, it lacks an environmental laboratory. From a health disparities perspective, despite classification as an upper middle-income economy, Dominica has a poverty level of 28.8% [25,28]. The key weather factors include hurricanes, heavy rainfall, African dust events, heat, and drought. The main climate-sensitive health concerns are vector-borne diseases (e.g., dengue), gastroenteritis, and food insecurity [29]. Building climate resiliency is a national priority. The health services have developed a strategy to mainstream climate change adaptation within the health system to include the development of a health vulnerability and adaptation assessment. There is ongoing development of a National Health Adaptation Plan, the establishment of the National Resilience Development Strategy, and participation in the PAHO/WHO Climate Smart Health Care Facilities project [10].

## Climate and health priorities

### Puerto Rico

Hurricane Maria’s impacts were central to discussions at the Puerto Rico workshop. Participants referred to the prolonged loss of electricity, electrical grid challenges, disruption of telecommunications, the shortage of physicians (heightened after the storm), constrained access to health care services, lack of critical care health facilities, stormwater management issues, social determinants of health, and heat tolerance in the local population. The following priorities for addressing climate and health were identified:

- Implement educational activities to increase public awareness about climate impacts on health.
- Convene workshops for decision-makers to facilitate a sustained multidisciplinary health and climate risk dialogue; promising topics include heat and respiratory issues.
- Identify climate-related decision support tools and best practices for integrating climate science in public health decision-making.
- Catalog existing data for weather, climate, respiratory conditions, heat-sensitive health conditions, and vector-borne diseases. From this, develop action-oriented strategies for each health concern.
- Develop stronger messaging around U.S. National Weather Service (NWS) heat products. Test and augment existing heat-related tools from the medical profession to support community efforts.
- Develop an online climate and health platform to improve information and data accessibility for decision-makers.

### Dominica

Hurricane Maria dominated discussion at the Dominica workshop. Participants cited the storm’s damage to health facilities and commodities, and elevated rates of food insecurity. They highlighted the vulnerability of individuals with chronic non-communicable diseases and the challenges of disease management during and after a disaster due to electricity loss and transportation and communication disruptions. The following were proposed as priorities to address climate and health:

- Create education opportunities for the health and climate workforce.
- Disseminate public service announcements on climate and weather risks that highlight health impacts and preventive measures.
- Implement communication and early warning systems to disseminate information on weather and climate-related risks and precautionary measures to be taken by residents and public health professionals.
- Modify existing weather and climate reports to be more accessible and user-friendly for public health practitioners.
- Develop predictive models for climate-sensitive health conditions.
- Establish stations to collect environmental data, including heat, rainfall, and air quality.
- Establish environmental health laboratory facilities.
- Modify neighborhood-level planning maps to geospatially identify health service areas.
- Leverage existing tools to better assess mental health and psychosocial issues in the context of disasters.

## Building awareness about climate and health

The stakeholder workshops led us to implement educational activities to increase awareness about the intersections between climate and health. In Puerto Rico, we hosted a workshop for health and weather and climate professionals on heat and hurricanes (February 2020). This workshop led to a collaboration between project members, a Puerto Rican science education organization (EcoExploratorio), academia, and the NWS in Puerto Rico. During the NWS’ heat awareness week in May 2020, EcoExploratorio organized 16 presentations on heat impacts and risk, climate change and heat, and co-occurrence of heat and hurricanes. We also organized 2 webinars for Caribbean-based environmental health professionals. The first webinar (August 2020) focused on linkages between climate, the COVID-19 pandemic, and non-communicable diseases, while the second (August 2021) was on climate services for health in the Caribbean.

Presenting environmental scan results at the stakeholder workshops was a catalyst for identifying priority actions. These workshops brought together representatives from sectors that do not often collaborate, revealing gaps that may otherwise have remained obscure. Stakeholders at both workshops identified benefits of more interdisciplinary collaboration and resources for information generation and dissemination through cross-sector, accessible platforms. While both workshops highlighted needs for improved analytics, data-sharing, and decision-support tools, they also revealed a lack of knowledge of how climate and health overlap. With an increasing emphasis placed on new information tools from organizations and networks, such as the World Meteorological Organization and the Global Framework for Climate Services [30], climate and health strategies in Caribbean SIDS should not overlook gaps in knowledge about the connections between climate and health sector impacts.
